# Effect of inclined magnetic field on radiative heat and mass transfer in chemically reactive hybrid nanofluid flow due to dual stretching

**DOI:** 10.1038/s41598-023-34871-9

**Published:** 2023-05-15

**Authors:** Mubashar Arshad, Fahad M. Alharbi, Ali Hassan, Qusain Haider, Abdullah Alhushaybari, Sayed M. Eldin, Zubair Ahmad, Laila A. Al-Essa, Ahmed M. Galal

**Affiliations:** 1 Department of Mathematics, University of Gujrat, Gujrat, 50700 Pakistan; 2 Department of Mathematics, Al-Qunfudah University College, Umm Al-Qura University, Mecca, Saudi Arabia; 3 Department of Mathematics, College of Science, Taif University, P.O. Box 11099, Taif, 21944 Saudi Arabia; 4 Center of Research, Faculty of Engineering, Future University in Egypt, New Cairo, 11835 Egypt; 5 Unit of Bee Research and Honey Production, Faculty of Science, King Khalid University, P.O. Box 9004, Abha, 61413 Saudi Arabia; 6 Applied College, Mahala Campus, King Khalid University, P.O. Box 9004, Abha, 61413 Saudi Arabia; 7 Department of Mathematical Sciences, College of Science, Princess Nourah bint Abdulrahman University, P.O. Box 84428, Riyadh, 11671 Saudi Arabia; 8 Department of Mechanical Engineering, College of Engineering in Wadi Alddawasir, Prince Sattam bin Abdulaziz University, Wadi Alddawasir, Saudi Arabia; 9 Production Engineering and Mechanical Design Department, Faculty of Engineering, Mansoura University, P.O. 35516, Mansoura, Egypt

**Keywords:** Fluid dynamics, Applied mathematics

## Abstract

This research analyzes the three-dimensional magneto hydrodynamic nanofluid flow through chemical reaction and thermal radiation above the dual stretching surface in the presence of an inclined magnetic field. Different rotational nanofluid and hybrid nanofluids with constant angular velocity ${\upomega }^{*}$ for this comparative study are considered. The constitutive relations are used to gain the equations of motion, energy, and concentration. This flow governing extremely non-linear equations cannot be handled by an analytical solution. So, these equations are transformed into ordinary differential equalities by using the similarity transformation and then handled in MATLAB by applying the boundary values problem practice. The outcomes for the considered problem are accessed through tables and graphs for different parameters. A maximum heat transfer amount is observed in the absence of thermal radiation and when the inclined magnetic field and axis of rotation are parallel.

## Introduction

Nanofluid was first devised by Choi and Eastman^1^ in the last decade of the 20th century. They revealed that when a nanoparticle having good thermal conductivity is dispersed into a base fluid, the thermal conductivity of the formed solution exceptionally increases. They remarked that pumping power for heat transport in a heat exchanger considerably declines when the nanofluid is utilized as an working fluid in a heat exchanger instead of conventional working fluids like water, oils, and ethylene glycol. Further, when two nanoparticles of nano-meter are distributed into a host fluid, the formed mixture is known as a hybrid nanofluid^2^. Hayat and Nadeem^3^ elaborated on the hybrid nanofluid for heat transfer improvement for rotating flow using silver and copper oxide nanoparticles. Shah and Ali^4^ provided a comprehensive review of hybrid nanofluids and their applications which involve industrial applications, like wire drawing, coolant in engines of automobiles, nuclear reactors, micro-chips in computers, hot rolling, cancer therapeutics, glass fiber production, as a detergent, etc. Yasmin et al.^5^ experimentally explored hybrid nanofluids for solar and thermal energy storage uses. Maddammasetty and Sireesha^6^ used thermal systems to elaborate the heat transport applications of hybrid nanofluids. Numerous researchers have investigated the application of nano and hybrid nanoliquids in recent years. Some of the recently studied nano and hybrid nanofluids are given for knowledge gains^7–12^. There is vast room for research in this regard.

Ghasemi et al.^13^ studied nanofluid flow over the stretched surface with radiation and magnetic field effect using a novel spectral relaxation method. They remarked that high levels of magnetic field dramatically affect temperature and concentration profile. Sharma et al.^14^ analyzed graphene Maxwell nanofluid past stretching surface. Viscous dissipation and unsteadiness reduce heat transfer rates. Hussain et al.^15^ studied three-dimensional nanofluid flow with a magneto effect. Zinc nano-particle embedded nanofluid displayed a higher rate of heat transmission with an increment in magnetization force. Arshad and Hassan^16^ explored hybrid nanofluids between rotating systems. They discovered that when two nanoparticles are present in a host fluid, the amount of heat transfer enhances. Hassan et al.^17^ explored hybrid nanofluid for prescribed wall temperature cases with thermal radiation. Reduced drag and lift coefficients are obtained when the silver nanoparticle is used with a single-wall carbon nanotube. Hady et al.^18^ elaborated on the radiation's influence on heat transfer in viscous movement across a stretching surface. TiO_2_-embedded nanofluids have higher cooling power as compared to other examined nanofluids. Ali et al.^19^ studied electro-magneto-hydrodynamic nanofluid flow with variable heat fluxes. They remarked that the electric field directly influences the temperature profile. In recent years, Hassan^20^, Hussain^21^, Sheikholeslami^22^, Masood and Farooq^23^ and Masood^24^ have explored hybrid nanofluid in the presence of different body forces.

There are two types of chemical reactions specifically, irreversible, and reversible chemical reactions. Irreversible chemical reactions are chemical reactions that can not return to their initial stage. Whereas the reversible chemical reaction can return to its initial stage in the presence of a catalyst^25^. Recently, different-order chemical reactions are explored by researchers to investigate their impact on flow regimes. Anjum et al.^26^ investigated binary chemical reaction effect fluid flow with double stratification impacts. They found out that skin friction enhanced with an augmentation in magnetizing force. Abbas et al.^27^ explained the influence of chemical reactions on heat transmission in third-grade fluid over an exponential stretching surface. Temperature enhances with a rise in the level of chemical response they remarked. Elattar et al.^28^ investigated the hybrid nanoliquid movement with hall current and chemical reaction effects. Species transportation enhanced with increment in chemical reaction they noted. Krisna et al.^29^ explained the chemical consequence and radiation effect on convective stream with sucking and heat-making effects. The amount of heat transfer is highly influenced by the chemical reaction and magnetic force. Recently, second-order and higher-order chemical reactions have been investigated by numerous researchers^30–32^.

In the 20th span, Prandtl^33^ pioneered the idea of boundary layer flow in fluid dynamics. The boundary layer is the layer of the fluid that forms in the surrounding area of the surface bounding the fluid. Most common examples of boundary layer flows include, near the earth's surface, the interior of water pipes, and inside the blood vessels^34^. Khan and Pop^35^ investigated the boundary layer flow past a stretching surface. They remarked that the Nusselt number is minimizing the phenomenon of thermophoresis, Brownian, Prandtl, and Lewis numbers. Crane et al.^36^ analyzed flow past the stretching surface, Bongar^37^ examined nanofluid flow past the stretching surface, Erickson^38^ explored heat and mass transmission with suction effect on a continuous level plate, and Sakiadis^39^ investigated axisymmetric flow in two-dimensional form for boundary layer flow behavior.

Chen and Stroble^40^ explored the Buoyancy force on the boundary layer flow on an continuously moving plate. Takhar and Nath^41^ discussed three-dimensional flow due to stretching surfaces. Wang et al.^42^ examined viscous flow above a stretchable surface together with slip and suction effects. They stated that the width of the boundary layer enhanced with minimal mass suction effect Mehmood and Ali^43^ explored analytical solutions of viscous flow with heat transfer. Shahzad et al.^44^ examined heat transfer due to stretching surfaces using nanoparticles with the MHD effect. They remarked that rotation enhances the skin friction of rotating nanofluids. Hassan et al.^45^ studied linear and nonlinear radiation effects on heat and mass transmission in a hybrid nanoliquid due to a stretchable surface. Hussain et al.^46^ discussed the magnetic and nonlinear thermal radiative effects on three-dimensional movement due to stretching surfaces. Arshad et al.^47^ examined magneto-hydrodynamic flow with chemical effect above the exponential stretching surface. Khan et al.^48^ studied heat and mass transmission in Burger's nanofluid with magnetization and chemical reaction over an exponential stretchable surface. Numerous researchers have explored stretching surfaces for heat transfer and analysis of fluid flow^49–53^.

The above-conducted literature review suggests that numerous researchers have investigated the flow past the stretching surface. Arshad^53^ explored the thermophoresis and Brownian motion with thermal radiative effect and uniform magnetic field. Arshad^47^ investigated the chemical reaction effect over an exponential stretching surface. The novelty of this paper is to investigate the comparative dynamics of rotating water-based nano and hybrid nanofluids over dual stretching surfaces implanted in a permeable medium with radiative heat and mass transfer. Arshad^47,53^ examined a uniform magnetic field, whereas in this study we have incorporated an inclined magnetic field with a chemical reaction. The prevailing equations are changed into the ordinary differential equation by employing a similarity transformation and attempted MATLAB by utilizing the boundary value problem method. The tolerance is set to be ${10}^{-6}$ for obtained solutions. The flow behavior and characteristics of magneto hydrodynamic nanofluid and hybrid nanofluid are comparatively presented through tables and graphs for different parameters. By increasing parameters, the skin friction, Nusselt number, and Sherwood number are evaluated. This relative study helps to answer the following main research questions.

1. What is the influence of rotation parameters on velocity, temperature, and concentration profiles?
2. How does the increasing behavior of the magnetic force and porous medium parameter affect the primary and secondary velocity profile?
3. Do the increasing behavior of the radiation parameter and Prandtl number give the increased heat transmission rate and minimum skin friction?
4. How does chemical reaction, Lewis number change the temperature and concentration profiles?
5. What is the changing behavior on skin frictions along the $x-axis$, $y-axis,$ Nusselt number as well as the Sherwood number of different parameters?

## Basic equations

The basic flow governing equations for viscid incompressible liquid over the permeable surface in the existence of Rosseland radiation, and chemical reaction.

**Continuity equation:**

1 $$\nabla .V=0.$$

**Momentum equation:**

2 $$\rho \left[{ V}_{t}+\left(V.\nabla \right)V\right]=-\nabla \mathrm{P}+\rho g+\mu {\nabla }^{2}V-\left[\frac{\mu }{{K}_{1}}\right]V.$$

**Energy equation:**

3 $${\rho C}_{p}\left[\frac{\partial T}{\partial t}+\left(V.\nabla \right)T\right]={k}_{f}{\nabla }^{2}T-\left[\frac{\partial }{\partial z} {q}_{r}\right].$$

**Concentration equation:**

4 $${C}_{t}+\left(V.\nabla \right)C={D}_{B}{\nabla }^{2}C+\frac{{D}_{T}}{{T}_{\infty }}{\nabla }^{2}T-{K}_{r}\left(C-{C}_{\infty }\right).$$

## Mathematical formulation of problem

**Figure 1:**
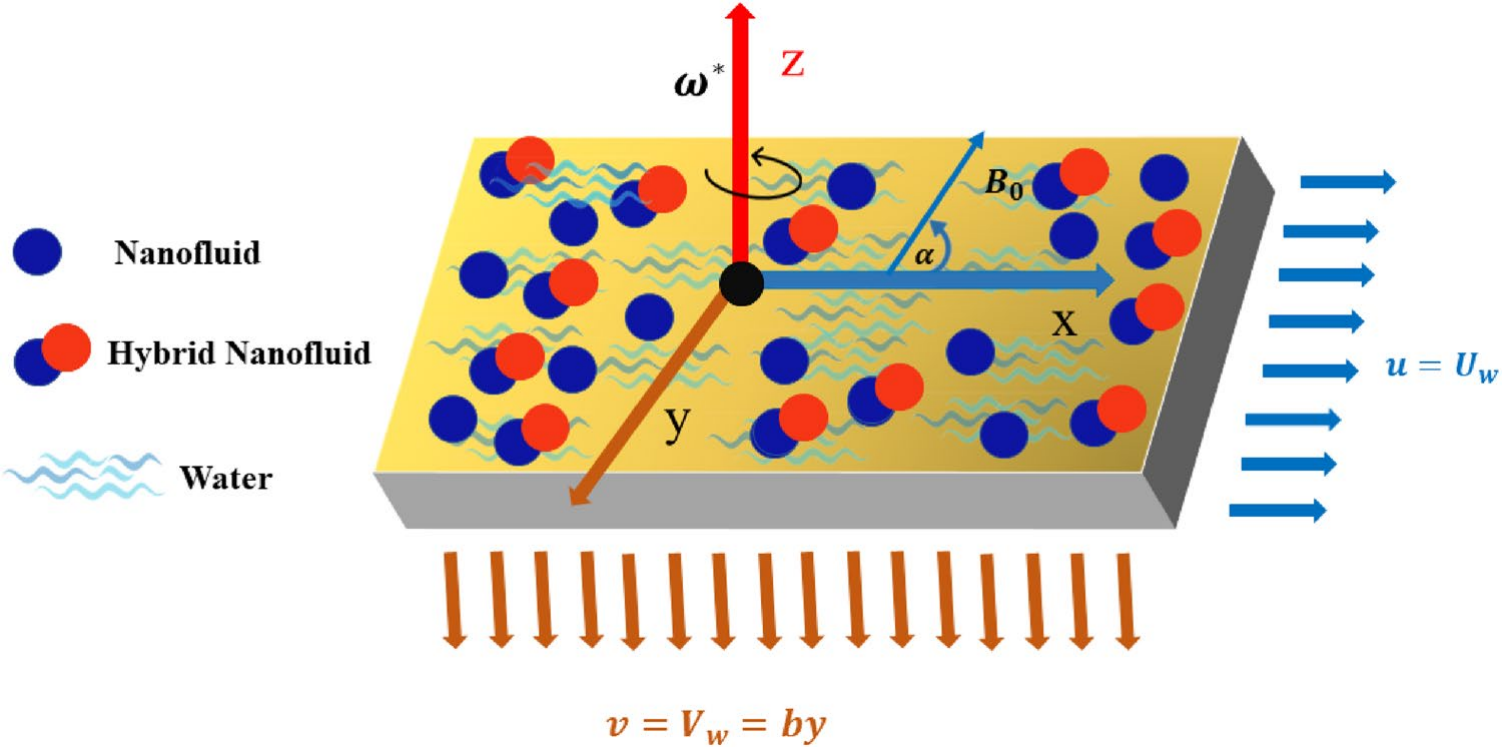
Flow configuration of the problem.

Consider an incompressible, steady, three-dimensional water-based nanofluid flowing above a porous stretchable sheet. A cartesian coordinate system $\left(x,y,z\right)$ is considered to discuss the problem physically. The x-axis is taken in the horizontal direction, the z-axis is upward and the y-axis is perpendicular to both other axes. An inclined magnetic field ${B}_{0}$ in the z-axis direction with angle $\alpha$ is acting. The nanofluid is rotating at a constant speed with ${\upomega }^{*}$ along the z-axis, chemical reaction and, thermal radiation are considered. The flow is induced by a stretching sheet with speeds ${U}_{w}=ax$ and ${V}_{w}=by$ in the x-direction and the y-direction (see Fig. 1). Nanofluid and hybrid nanofluid are considered for this comparative study. The governing equation along with these considerations takes the following form^53^:

**Equation of continuity** ^53^ **:**

5 $${u}_{x} + {v}_{y} + {w}_{z} = 0,$$

**Momentum equations along the** ${\varvec{x}}$ **and** ${\varvec{y}}$ **axis** ^53^ **:**

6 $$\left(u {u}_{x} + v {u}_{y}+w {u}_{z}-2{\omega }^{*}v\right)=\frac{{\mu }_{hnf}}{{\rho}_{hnf}}\left({u}_{xx}+{u}_{yy}+{u}_{zz}\right)-\frac{{\sigma }_{hnf}}{{\rho }_{hnf} }{{B}_{0}}^{2}{sin}^{2}\left(\alpha \right)u-\frac{{\mu }_{hnf}}{{\rho }_{hnf}}\frac{u}{{k}_{o}},$$

7 $$\left(u{ v}_{x} + v { v}_{y}+w { v}_{z}-2{\omega }^{*}u\right)=\frac{{\mu }_{hnf}}{{\rho }_{hnf}}\left({ v}_{xx}+{ v}_{yy}+{v}_{zz}\right)-\frac{{\sigma}_{hnf}}{{\rho }_{hnf} }{{B}_{0}}^{2}{sin}^{2}\left(\alpha \right)v-\frac{{\mu }_{hnf}}{{\rho }_{hnf} }\frac{v}{{k}_{o}},$$

**Energy equation without** ${{\varvec{q}}}_{{\varvec{r}}}$ **relation** ^53^ **:**

8 $$u{ T}_{x} + v { T}_{y}+w{ T}_{z}={\alpha }_{1}\left( { T}_{xx}+{ T}_{yy}+{ T}_{zz}\right)+\tau \left[{D}_{B}\left( { C}_{x}.{ T}_{x}+{ C}_{y}.{ T}_{y}+{ C}_{z}.{ T}_{z}\right)+\frac{{D}_{T}}{{T}_{\infty }}\left( { T}_{xx}+{ T}_{yy}+{ T}_{zz}\right)\right]-\frac{1}{{\left({\rho C}_{p}\right)}_{hnf}}\frac{\partial }{\partial z}\left({q}_{r}\right)$$

**Concentration equation** ^53^ **:**

9 $$u{ C}_{x} + v { C}_{y}+w{ C}_{z}={D}_{B}\left( { C}_{xx}+{ C}_{yy}+{ C}_{zz}\right)+\frac{{D}_{T}}{{T}_{\infty }}\left({ T}_{xx}+{ T}_{yy}+{ T}_{zz}\right)-\frac{1}{{\left({\rho C}_{p}\right)}_{hnf}}{K}_{r}\left(C-{C}_{\infty }\right)$$

Here $u,v$, and $w$ are velocity components in the $x,y$, and $z$ directions respectively. $T$ and $C$ are temperature and concentration.${k}_{0}, {q}_{r},{g}^{*}, {B}_{0}, and {K}_{r}$ is the porosity, radiation, gravitational acceleration, magnetic field, and chemical reaction respectively.${\rho }_{hnf},{\mu }_{hnf}, {\mathrm{\alpha }}_{1}$ is the density, viscosity, and thermal diffusivity of hybrid nanofluid. By applying the Rosseland approximation, the radiative heat flux ${q}_{r}$ is defined by^53^:

10 $${q}_{r}=-\frac{4{\sigma }^{*}}{3{k}_{1}}\frac{{\partial T}^{4}}{\partial z}.$$

Here ${\sigma }^{*}$ is the Stefan-Boltzmann coefficient and ${k}_{1}$ is the mean absorption constant. Assuming that there is not much of a temperature differential within the flow, the expression of the term ${T}^{4}$ by using Taylor series expansion is expanded as follows:

11 $${T}^{4}={{T}^{4}}_{\infty }+{4{T}^{3}}_{\infty }\left(T-{T}_{\infty }\right)+{6{T}^{2}}_{\infty }{\left(T-{T}_{\infty }\right)}^{2}+\dots$$

Consequently, by ignoring higher order terms above the first order in $\left(T-{T}_{\infty }\right)$, we get.

12 $${T}^{4}=4{{T}^{3}}_{\infty }T-3{{T}^{4}}_{\infty }$$

By using Eqs. (11) and (12)

13 $$\frac{{\partial q}_{r}}{\partial z}=-\frac{16{\sigma }^{*}{{T}_{\infty }}^{3}}{{3k}_{1}}\frac{{\partial }^{2}T}{{\partial z}^{2}}$$

Therefore, the energy equation takes the form.

14 $${u T}_{x} + v { T}_{y}+w{ T}_{z}={\alpha }_{1}\left( { T}_{xx}+{ T}_{yy}+{ T}_{zz}\right)+\tau \left[{D}_{B}\left({ C}_{x}.{ T}_{x}+{ C}_{y}.{ T}_{y}+{ C}_{z}.{ T}_{z}\right)+\frac{{D}_{T}}{{T}_{\infty }}\left( { T}_{xx}+{ T}_{yy}+{ T}_{zz}\right)\right]+\frac{16{\sigma }^{*}{{T}_{\infty }}^{3}}{{3k}_{1}{\left({\rho C}_{p}\right)}_{hnf}}{ T}_{zz}$$

The corresponding boundary conditions are:

15 $$\left.\begin{array}{c}u={U}_{w}=ax, v={V}_{w}=by, w=0, T={T}_{w}, C={C}_{w}, at z=0 \\ u\to 0, v\to 0, T\to {T}_{\infty }, C\to {C}_{\infty }, as z\to \infty \end{array}\right\}$$

### Similarity transformation

We define the following similarity transformation to transform the equation which are tackled numerically.

16 $$\begin{aligned} & u=ax{p}^{^{\prime}}\left(\eta \right), \quad v=ay{q}^{^{\prime}}\left(\eta \right), \quad w=-\sqrt{a{v}_{f} }\left\{p\left(\eta \right)+q\left(\eta \right)\right\}, \quad r\left(\eta \right)\left({T}_{w}-{T}_{\infty }\right)=T-{T}_{\infty }, \quad \\ & s\left(\eta \right)\left({C}_{w}-{C}_{\infty }\right)=C-{C}_{\infty }, \quad \eta =z\sqrt{\frac{a}{{v}_{f}}.}\end{aligned}$$

Here differential is w.r.t $\eta$. By using this similarity transformation Eq. (16), the Eq. (5) is satisfied. Equations (6), (7), (9) and Eq. (14) takes the following transformed form for hybrid nanofluid.

17 $${p}^{{^{\prime}}{^{\prime}}{^{\prime}}}\left(\eta \right)={B}_{1}*\left\{{{p}^{^{\prime}}\left(\eta
\right)}^{2}\left({p}^{^{\prime}}\left(\eta \right)+{q}^{^{\prime}}\left(\eta \right)\right)-2 \lambda \delta{q}^{^{\prime}}\left(\eta \right)+Z {p}^{^{\prime}}\left(\eta \right)+{M}^{2}{sin}^{2}\left(\alpha
\right){p}^{^{\prime}}{B}_{5}\right\}*{K}_{2}$$

18 $${q}^{{^{\prime}}{^{\prime}}{^{\prime}}}\left(\eta \right)={B}_{1}*\left\{{{q}^{^{\prime}}\left(\eta \right)}^{2}\left({p}^{^{\prime}}\left(\eta \right)+{q}^{^{\prime}}\left(\eta \right)\right)-2\frac{\lambda }{\delta }
{p}^{^{\prime}}\left(\eta \right)+Z {q}^{^{\prime}}\left(\eta \right)+{M}^{2}{sin}^{2}\left(\alpha \right){q}^{^{\prime}}{B}_{5}\right\}*{K}_{2}$$

19 $${r}^{{^{\prime}}{^{\prime}}}=-{\left(1+\frac{4}{3{B}_{3}}*\pi \right)}^{-1}*\left[Pr*{B}_{2}*{r}^{^{\prime}}\left(p\left(\eta \right)+q\left(\eta \right)\right)+{r}^{^{\prime}}{s}^{^{\prime}}{N}_{b}+{{r}^{^{\prime}}}^{2}{N}_{t}\right]$$

20 $${s}^{{^{\prime}}{^{\prime}}}=Le\left(\left(p+q\right){r}^{^{\prime}}+\frac{{N}_{t}}{{N}_{b}}{r}^{{^{\prime}}{^{\prime}}}-s{K}_{c}\right)$$

The non-dimensional quantities ${B}_{1},{ B}_{2}, {B}_{3},{ B}_{4}$ and ${K}_{2},$ are double nanoparticle relations (defined in Table 1), and $\lambda , \, \delta , \, Z, \, \epsilon , \, M, \, Pr, \, \pi , \, Le, \, {N}_{t}, \, \tau , \, {N}_{b},$ and ${K}_{c}$ are defined as

**Table 1:** Thermophysical relations of nanoparticles and base fluid^53^.

Properties	Nanofluid
Density	${\rho }_{nf}=(1-{(\phi }_{1})){\rho }_{f}+{\phi }_{1}{\rho }_{s1}, {A}_{1}=\frac{{\rho }_{nf}}{{\rho }_{f}}$
Dynamic viscosity	${\mu }_{nf}=\frac{{\mu }_{f}}{{[1-\left({\phi }_{1}\right)]}^{5/2}}={K}_{1}$
Heat capacity	${\left(\rho {C}_{p}\right)}_{nf}=\left[1-\left({\phi }_{1}\right)\right]{\left(\rho {c}_{p}\right)}_{f}+{{\phi }_{1}(\rho {c}_{p})}_{s1}, {A}_{2}=\frac{{\left(\rho {C}_{p}\right)}_{nf}}{{\left(\rho {C}_{p}\right)}_{f}}$
Thermal conductivity	$\frac{{k}_{nf}}{{k}_{f}}=\frac{{\phi }_{1}{k}_{s1}+2{k}_{f}{\phi }_{1}+2{{\phi }_{1}}^{2}{k}_{s1}-2{{\phi }_{1}}^{2}{k}_{f}}{{\phi }_{1}{k}_{s1}+2{k}_{f}{\phi }_{1}-{{\phi }_{1}}^{2}{k}_{s1}+{{\phi }_{1}}^{2}{k}_{f}}, {A}_{3}=\frac{{k}_{nf}}{{k}_{f}}$
Electrical conductivity	$\frac{{\sigma }_{nf}}{{\sigma }_{f}}=1+\frac{3\left({\sigma }_{s1}-{\sigma }_{f}\right)}{\left({\sigma }_{s1}+2{\sigma }_{f}\right)-\left({\sigma }_{s1}-{\sigma }_{f}\right){\phi }_{1}}, {A}_{5}=\frac{{\sigma }_{nf}}{{\sigma }_{f}}$
Thermal expansion	${\left(\rho {B}_{t}\right)}_{nf}=(1-{(\phi }_{1})){\rho {B}_{t}}_{f}+{\phi }_{1}{\rho {B}_{t}}_{s1}, {A}_{4}=\frac{{\left(\rho {B}_{t}\right)}_{nf}}{{\left(\rho {B}_{t}\right)}_{f}}$

Properties	Hybrid nanofluid
Density	${\rho }_{hnf}=(1-{(\phi }_{1}+{\phi }_{2})){\rho }_{f}+{\phi }_{1}{\rho }_{s1}+{\phi }_{2}{\rho }_{s2}, {B}_{1}=\frac{{\rho }_{hnf}}{{\rho }_{f}}$
Dynamic viscosity	${\mu }_{hnf}=\frac{{\mu }_{f}}{{[1-\left({\phi }_{1}+{\phi }_{2}\right)]}^{5/2}}={K}_{2}$
Heat capacity	${\left(\rho {C}_{p}\right)}_{hnf}=\left[1-\left({\phi }_{1}+{\phi }_{2}\right)\right]{\left(\rho {c}_{p}\right)}_{f}+{{\phi }_{1}(\rho {c}_{p})}_{s1}+{{\phi }_{2}(\rho {c}_{p})}_{s2}, {B}_{2}=\frac{{\left(\rho {C}_{p}\right)}_{hnf}}{{\left(\rho {C}_{p}\right)}_{f}}$
Thermal conductivity	${b}_{1}={\phi }_{1}{k}_{s1}+{\phi }_{2}{k}_{s2}+2{k}_{f}\left({\phi }_{1}+{\phi }_{2}\right)+2\left({\phi }_{1}+{\phi }_{2}\right)\left({\phi }_{1}{k}_{s1}+{\phi }_{2}{k}_{s2}\right)-2{\left({\phi }_{1}+{\phi }_{2}\right)}^{2}{k}_{f}$ ${b}_{2}={\phi }_{1}{k}_{s1}+{\phi }_{2}{k}_{s2}+2{k}_{f}\left({\phi }_{1}+{\phi }_{2}\right)-\left({\phi }_{1}+{\phi }_{2}\right)\left({\phi }_{1}{k}_{s1}+{\phi }_{2}{k}_{s2}\right)+{\left({\phi }_{1}+{\phi }_{2}\right)}^{2}{k}_{f}$ $\frac{{k}_{hnf}}{{k}_{f}}=\frac{{b}_{1}}{{b}_{2}}={B}_{3}, {B}_{3}=\frac{{k}_{hnf}}{{k}_{f}}$
Thermal expansion	${\left(\rho {B}_{t}\right)}_{hnf}=(1-{(\phi }_{1}+{\phi }_{2})){\left(\rho {B}_{t}\right)}_{f}+{\phi }_{1}{\left(\rho {B}_{t}\right)}_{s1}+{\phi }_{2}{\left(\rho {B}_{t}\right)}_{s2}, {B}_{4}=\frac{{\left(\rho {B}_{t}\right)}_{hnf}}{{\left(\rho {B}_{t}\right)}_{f}}$
Electrical conductivity	$\frac{{\sigma }_{hnf}}{{\sigma }_{f}}=1+\frac{3\left[\frac{{\sigma }_{s1}{\phi }_{1}-{\sigma }_{s2}{\phi }_{2}}{{\sigma }_{f}}-{(\phi }_{1}+{\phi }_{2})\right]}{\left(2+\frac{{\sigma }_{s1}+{\sigma }_{s2}}{{\sigma }_{f}}\right)-\left[\frac{{\sigma }_{s1}{\phi }_{1}-{\sigma }_{s2}{\phi }_{2}}{{\sigma }_{f}}\right]+{(\phi }_{1}+{\phi }_{2})}, {B}_{5}=\frac{{\sigma }_{hnf}}{{\sigma }_{f}}$

21 $$\begin{aligned} \lambda &=\frac{{\omega }^{*}}{a}, \quad \delta =\frac{y}{x}, \quad Z=\frac{{\mu }_{hnf}}{{a{\rho }_{hnf}k}_{o}},\quad M= \sqrt{\frac{{\sigma }_{f}{{B}_{0}}^{2}}{a{\rho }_{f}}},\quad Pr=\frac{{v}_{f}}{{k}_{f}}, \pi =\frac{4{\sigma }^{*} {{T}_{\infty }}^{3}}{{k}_{1} {k}_{f}}, \quad Le= \frac{{D}_{B}}{{v}_{f}}, \quad \tau =\frac{{\left(\rho Cp\right)}_{s}}{{\left(\rho Cp\right)}_{f}}, \quad {N}_{t}=\frac{{\left(\rho Cp\right)}_{s}{D}_{B}\left({C}_{w}-{C}_{\infty }\right)}{{\left(\rho Cp\right)}_{f} {v}_{f}} , \\& {N}_{b} =\frac{{\left(\rho Cp\right)}_{s}{D}_{T}\left({T}_{w}-{T}_{\infty }\right)}{{\left(\rho Cp\right)}_{f}{T}_{\infty } {v}_{f}}, \quad {K}_{c}=\frac{{K}_{r}}{a}.\end{aligned}$$

Here $${\mathrm{Gr}}_{x}=\frac{{g}^{*}{\left(\rho {B}_{t}\right)}_{hnf}}{{{v}_{f}}^{2}}\left(T-{T}_{\infty }\right) {x}^{3}, {Re}_{x}=\frac{{u}_{w}\left(x\right)}{{v}_{f}}$$and $${\mathrm{Gr}}_{y}=\frac{{g}^{*}{\left(\rho {B}_{t}\right)}_{hnf}}{{{v}_{f}}^{2}}\left(T-{T}_{\infty }\right) {y}^{3}, {Re}_{y}=\frac{{v}_{w}(y)}{{v}_{f}}$$

The modified boundary conditions are as follows:

22 $$\left.\begin{array}{c}p=0, {p}^{^{\prime}}=1, q=0, {q}^{^{\prime}}=\gamma , r=1, s=1, at \eta =0\\ {p}^{^{\prime}}\to 0, {q}^{^{\prime}}\to 0, r\to 0, s\to 0, as \eta \to \infty \end{array}\right\}$$

Here $\gamma =\frac{b}{a}$ is the dimensionless stretching ratio.

### Physical quantities of interest

The most significant physical quantities of importance from an engineering perspective are the skin friction coefficients ${Cf}_{x}$, ${Cf}_{y}$, and Nusselt number $Nu$, which are defined as follows:

23 $${Cf}_{x}=\frac{{\tau }_{zx}}{{\rho }_{f}{{u}_{w}}^{2}}, {Cf}_{y}=\frac{{\tau }_{zy}}{{\rho }_{f}{{v}_{w}}^{2}}$$

Here ${\tau }_{zx}$ and ${\tau }_{zy}$ denote the shear stress along the stretched wall along the *x*-axis and *y*-axis and are defined as

24 $${\tau }_{zx}={\mu }_{hnf}{\left(\frac{\partial u}{\partial z}+\frac{\partial w}{\partial x}\right)}_{z=0} , {\tau }_{zy}={\mu }_{hnf}{\left(\frac{\partial v}{\partial z}+\frac{\partial w}{\partial y}\right)}_{z=0}$$

The dimensionless form of Eq. (18) is:

25 $${\left({Re}_{x}\right)}^{1/2} C{f}_{x}=\frac{{\mu }_{hnf}}{{\mu }_{f}} {p}^{{^{\prime}}{^{\prime}}}\left(0\right), {\left({Re}_{x}\right)}^{1/2} C{f}_{y}=\frac{{\mu }_{hnf}}{{\mu }_{f}} {q}^{{^{\prime}}{^{\prime}}}\left(0\right),$$

By using the temperature field to define the thermal diffusion rate as a Nusselt number:

26 $${Nu}_{x}=\frac{x{q}_{w}}{{k}_{f}({T}_{w}-{T}_{\infty })}, {q}_{w}=-{k}_{hnf}{\left(\frac{\partial T}{\partial z}\right)}_{z=0}+ {{(q}_{r})}_{w},$$

Or

27 $${Nu}_{x}=-\left({B}_{3}+\frac{4}{3}\pi \right){r}^{^{\prime}}\left(0\right),$$

By using the concentration field to define the mass transmission rate as Sherwood number:

28 $${Sh}_{x}=\frac{x{q}_{m}}{{D}_{m}\left({C}_{w}-{C}_{\infty }\right)}, {q}_{m}={D}_{m}{\left(\frac{\partial C}{\partial z}\right)}_{z=0}$$

By applying the resemblance transformation Eq. (16), the non-dimensional form of the Sherwood number takes the form:

29 $${Sh}_{x}=-{s}^{^{\prime}}\left(0\right).$$

The following Tables 1 and 2 show the thermophysical relations and values for the formulation of nanofluid and hybrid nanofluid.

**Table 2:** Thermophysical characteristics of the base fluid and nanoparticles^53^.

Physical properties	Electrical conductivity	Density	Specific heat	Thermal conductivity	Thermal expansion
Water	0.05	$997$	$4179$	$0.614$	$21\times {10}^{-5}$
$Copper \left({s}_{1}\right)$	$5.96\times {10}^{7}$	$8933$	$385$	$400$	$1.67\times {10}^{-5}$
$Aluminum oxide \left({s}_{2}\right)$	$6.27\times {10}^{-5}$	$3970$	$765$	$40$	$0.85\times {10}^{-5}$

Spherical-shaped nanoparticles are used having the shape effect $3$. Additionally, the volumetric concentration of the nanoparticle used is about 0.005% for each nanoparticle.

### Numerical scheme and validation

The boundary value problem technique is used. Highly accurate and effective numeric outcomes are obtained (Table 3) when we use this technique. Equations (17)–(20) which are highly non-linear are changed to first-order ODEs by using a new set of variables defined as follows.

**Table 3:** The valuation of the current numerical results with the literature.

$\lambda$	Present outcomes		Wang^54^		Nazar et al.^55^	
	*pʺ *(0)	*qʺ *(0)	*pʺ *(0)	*qʺ *(0)	*pʺ *(0)	*qʺ *(0)
$0.0$	$-1.0$	$0.0$	$-1.0$	$0.0$	$-1.0$	$0.0$
$0.5$	$-1.145$	$-0.569$	$-1.13$	$-0.51$	$-1.13$	$-0.51$
$1.0$	$-1.334$	$-0.888$	$-1.32$	$-0.83$	$-1.32$	$-0.83$
$2.0$	$-1.661$	$-1.328$	$-1.65$	$-1.28$	$-1.65$	$-1.28$

30 $$\begin{aligned} & {{y}_{3}}^{^{\prime}}={p}^{{^{\prime}}{^{\prime}}{^{\prime}}}, \quad {y}_{3}={p}^{{^{\prime}}{^{\prime}}}, \quad {y}_{2}={p}^{^{\prime}}, {y}_{1}=p, \quad {{y}_{6}}^{^{\prime}}={q}^{{^{\prime}}{^{\prime}}{^{\prime}}}, \quad {y}_{6}={q}^{{^{\prime}}{^{\prime}}}, \quad {y}_{5}={q}^{^{\prime}}, \quad {y}_{4}=q, \quad \\ & {{y}_{8}}^{^{\prime}}={r}^{{^{\prime}}{^{\prime}}}, \quad {y}_{8}={r}^{^{\prime}}, \quad {y}_{7}=r, \quad {{y}_{10}}^{^{\prime}}={s}^{{^{\prime}}{^{\prime}}}, \quad {y}_{10}={s}^{^{\prime}}, \quad {y}_{9}=s,\end{aligned}$$

The equation ${{y}_{3}}^{^{\prime}}, {{y}_{6}}^{^{\prime}},{{y}_{8}}^{^{\prime}}$ and ${{y}_{10}}^{^{\prime}}$ takes the following form:

31 $${{y}_{3}}^{^{\prime}}={B}_{1}*\left\{\left[{{y}_{2}}^{2}*\left({y}_{1}+{y}_{4}\right)\right]-\left[2* \lambda *\delta * {y}_{5}\right]+\left[Z*{y}_{2}\right]-\left[{\epsilon }_{x}*{y}_{7}*{B}_{4}\right]+\left[{M}^{2}*{y}_{2}*{sin}^{2}\left(\alpha \right)*{y}_{2}*{B}_{5}\right]\right\}*{K}_{2}$$

32 $${{y}_{6}}^{^{\prime}}={B}_{1}*\left\{\left[{{y}_{5}}^{2}*\left({y}_{1}+{y}_{4}\right)\right]-\left[2*\lambda *\frac{1}{\delta }* {y}_{2}\right]+\left[Z*{y}_{5}\right]-\left[{\epsilon }_{y}*{y}_{7}*{B}_{4}\right]+\left[{M}^{2}*{y}_{2}*{sin}^{2}\left(\alpha \right)*{y}_{5}*{B}_{5}\right]\right\}*{K}_{2}$$

33 $${{y}_{8}}^{^{\prime}}=-{\left(1+\frac{4}{3{B}_{3}}*\pi \right)}^{-1}\left\{\left[{y}_{8}*{y}_{10}*{N}_{b}+{{y}_{8}}^{2}*{N}_{t}\right]+\left[Pr*{B}_{2}*{y}_{8}*\left({y}_{1}+{y}_{4}\right)\right]\right\}$$

34 $${{y}_{10}}^{^{\prime}}= Le*\left(\left(\left(p+q\right)*{y}_{8}\right)+\left(\left(\frac{{N}_{t}}{{N}_{b}}\right)*{{y}_{8}}^{^{\prime}}\right)-\left({y}_{10}*{K}_{c}\right)\right)$$

The transformed boundary conditions changed into the subsequent form:

35 $$\left.\begin{array}{c}{y}_{1}=0, {y}_{2}=1, {y}_{4}=0, {y}_{5}=\gamma , {y}_{7}=1, at \eta =0\\ {y}_{2}\to 0, {y}_{5}\to 0, {y}_{7}\to 0, as \eta \to \infty \end{array}\right\}$$

The skin friction, Nusselt, and Sherwood numbers changed into the following form:

36 $${\left({Re}_{x}\right)}^{1/2} C{f}_{x}=\frac{{\mu }_{hnf}}{{\mu }_{f}}*{y}_{3}\left(0\right), {\left({Re}_{x}\right)}^\frac{1}{2} C{f}_{y}=\frac{{\mu }_{hnf}}{{\mu }_{f}}* {y}_{6}\left(0\right),$$

37 $${Nu}_{x}=-\left({B}_{3}+\frac{4}{3}\pi \right)*{y}_{8}\left(0\right), {Sh}_{x}=-{y}_{10}\left(0\right) .$$

## Results and discussion

The interpretation of the problem introduced in the preceding section is hybrid nanofluid. The outputs of the present problem are obtained separately for each nanofluid i.e., copper–water nanofluid, copper/aluminum oxide–water based hybrid nanofluid. The obtained effects of different parameters by utilizing the boundary value problem technique at MATLAB are described in this segment.

### Effect of rotation and stretching ratio parameter

**Figure 2:**
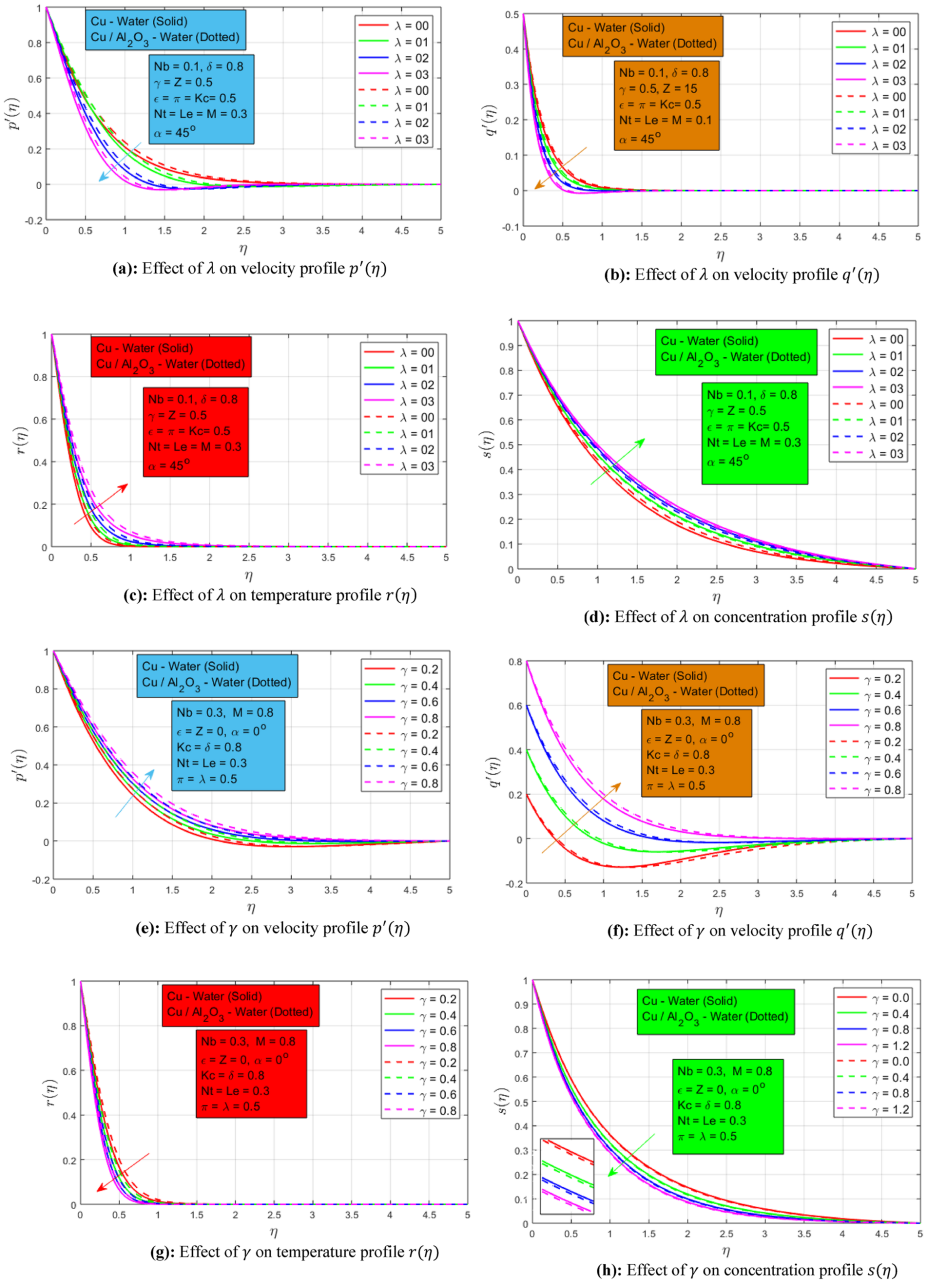
(**a**) Effect of $\lambda$ on velocity profile ${p}^{^{\prime}}(\eta )$, (**b**) Effect of $\lambda$ on velocity profile ${q}^{^{\prime}}(\eta )$, (**c**) Effect of $\lambda$ on temperature profile $r\left(\eta \right)$, (**d**) Effect of $\lambda$ on concentration profile $s\left(\eta \right)$, (**e**) Effect of $\gamma$ on velocity profile ${p}^{^{\prime}}(\eta )$, (**f**) Effect of $\gamma$ on velocity profile ${q}^{^{\prime}}(\eta )$, (**g**) Effect of $\gamma$ on temperature profile $r(\eta )$, (**h**) Effect of $\gamma$ on concentration profile $s(\eta )$.

The effect of rotation constraint on velocity constituents ${p}^{^{\prime}}\left(\eta \right),{q}^{^{\prime}}\left(\eta \right)$, temperature $r \left(\eta \right)$, and concentration $s\left(\eta \right)$ is presented in the following Fig. 2a–d respectively. The velocity profiles ${p}^{^{\prime}}\left(\eta \right)$ and ${q}^{^{\prime}}\left(\eta \right)$ are evident from the graph that it decays when rotation parameter $\lambda$ increases for nanofluid and hybrid nanofluid. At the start when $\lambda =0$ and $\lambda =1$, there is a minimum change in velocity profiles. When rotation increases by $\lambda =2$ and $\lambda =3,$ these profiles decay rapidly. The specific reason for the occurrence of this event is that rotation is a direct function of the angular velocity. Additionally, the minimum resistive Lorentz force is present when the rotation is increased. This effect expands the momentum layer in the primary direction whereas it is reduced in the secondary direction. An opposite behavior is noted for the impact of rotation constraint on temperature profile $r\left(\eta \right)$ and concentration profile $s\left(\eta \right)$ respectively. Temperature and concentration profiles increase when the rotation parameter increases. Here least rise is noted for the concentration profile as compared to the temperature profile. It is worth mentioning here that when rotation is enhanced the associated thermal boundary layer contracts for both nanofluid and hybrid nanofluids. Additionally, the presence of thermal radiation has a direct impact on the temperature profile. Moreover, it declines when the rotation and magnetization force is high. It is noted that under the high influence of rotation, the concentration profile declines rapidly. Chemical reactions aid the smooth movement of the concentration of nano-particles when the rotational motion of the fluid is augmented.

Figure 2e–h show the effect of stretching ratio constraints on velocities, temperature, and concentration profiles, respectively. When the stretching is enhanced velocity in the x-direction decline. Additionally, when stretching is enhanced the momentum layer associated with secondary velocity expands. Stretching has the opposite effect on the primary and secondary velocity profile. A slight difference has been observed in the dynamics of nano and hybrid nanoparticles. The temperature and concentration of both profiles show decreasing behavior for rising the stretching ratio parameter. It is fascinating to note that a high-temperature profile can be observed for a hybrid nanofluid, and a high-concentration profile can be observed for a single nanoparticle nanofluid. Additionally, the temperature boundary layer has contracted under the high increment of stretching ratio. Concentration profile decline with increment in the stretching ratio and the associated concentration layer contract due to the influence of chemical reaction.

### Effect of magnetic force and porosity

**Figure 3:**
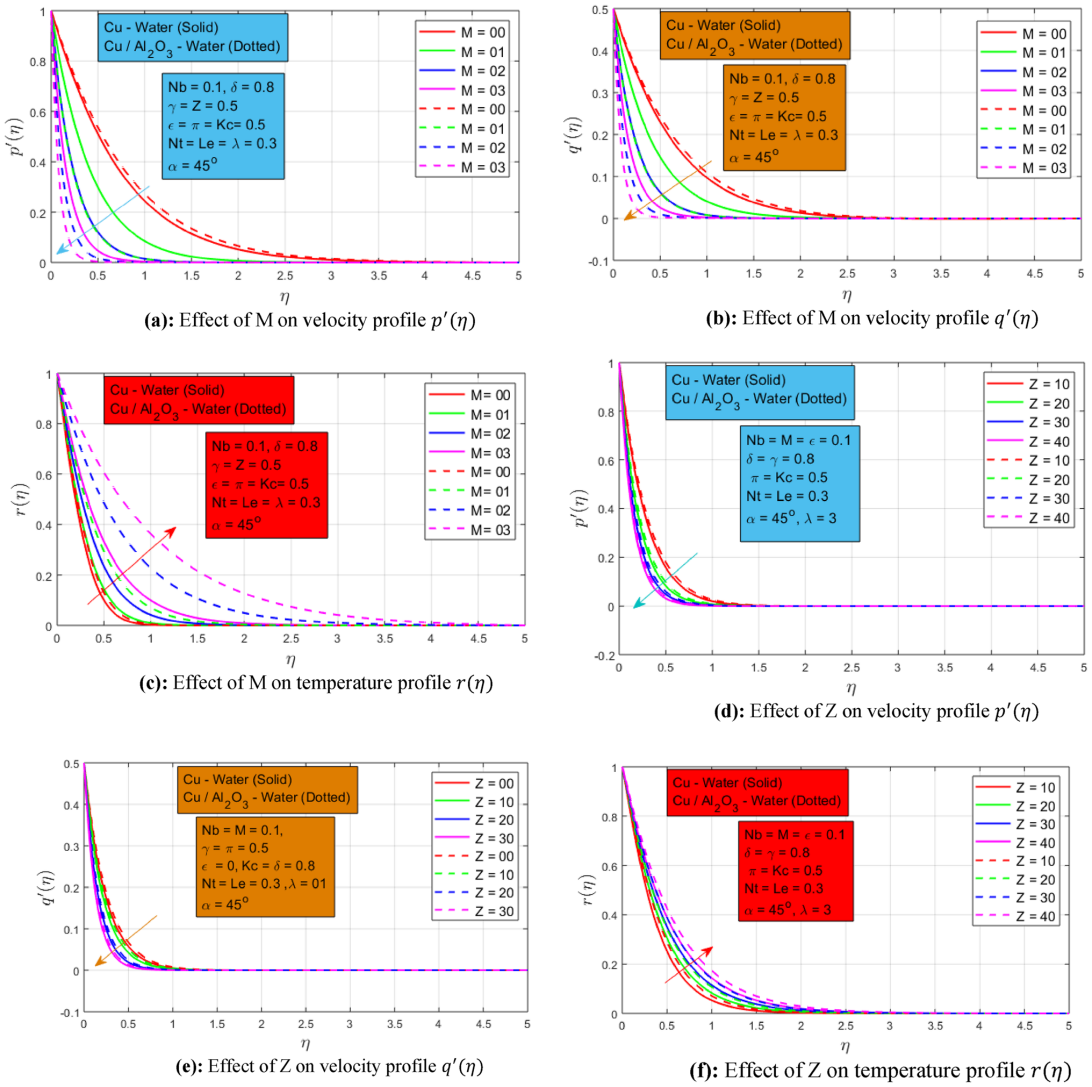
(**a**) Effect of M on velocity profile ${p}^{^{\prime}}(\eta )$. (**b**) Effect of M on velocity profile ${q}^{^{\prime}}(\eta )$. (**c**) Effect of M on temperature profile $r(\eta )$. (**d**) Effect of Z on velocity profile ${p}^{^{\prime}}(\eta )$. (**e**) Effect of Z on velocity profile ${q}^{^{\prime}}(\eta )$. (**f**) Effect of Z on temperature profile $r(\eta )$.

The following Figs. 3a–c present the effect of magnetic force $M$ on velocity profile ${p}^{^{\prime}}\left(\eta \right), {q}^{^{\prime}}(\eta )$, and temperature profile $r(\eta )$, respectively. In the non-existence of a magnetic force $(M=0),$ the fluid flow very smoothly, and when the magnetic field begins to work the fluid velocity profiles decays rapidly. This is owing to the Lorentz force acting on fluid which restricts the fluid to move and causes endurance and as a result, the velocity profiles decay under the rising behavior of the magnetic field constraint. Higher momentum border layer wideness is seen for hybrid nanoliquid in the non-existence of a magnetic force. An opposite behavior is presented for magnetic field constraint on temperature profile $r\left(\eta \right).$ The temperature profile has a direct relation with magnetic field constraint. As the magnetic force parameter grows, the temperature profile increases in the same manner and higher thermal frontier layer wideness is detected for hybrid nanofluid. The reason is that the Lorentz force restricts the flow which allows the fluid to transfer a higher amount of heat transmission. The change of porous medium parameter $Z$ on velocity profiles ${p}^{^{\prime}}(\eta )$, ${q}^{^{\prime}}(\eta )$, and temperature profile $r(\eta )$ is represented in Figs. 3d–f. The momentum boundary layer thickness has an inverse relation with the porous medium parameter. Both the velocity profiles ${p}^{^{\prime}}(\eta )$ and ${q}^{^{\prime}}(\eta )$ decreases when the permeability of the medium increases because the velocity is dependent on the porosity of the surface. There is an inverse relation between the permeability parameter and temperature profile $r(\eta )$. When the porosity of the surface increases the thermal boundary layer expands as an outcome the fluids conduct more heat. In both cases, greater momentum and thermal boundary layer are noted for the hybrid nanofluid as associated with the nanofluid. This shows the high performance of hybrid nanoparticles nanofluid.

### Effect of mixed convection and inclined magnetic field

**Figure 4:**
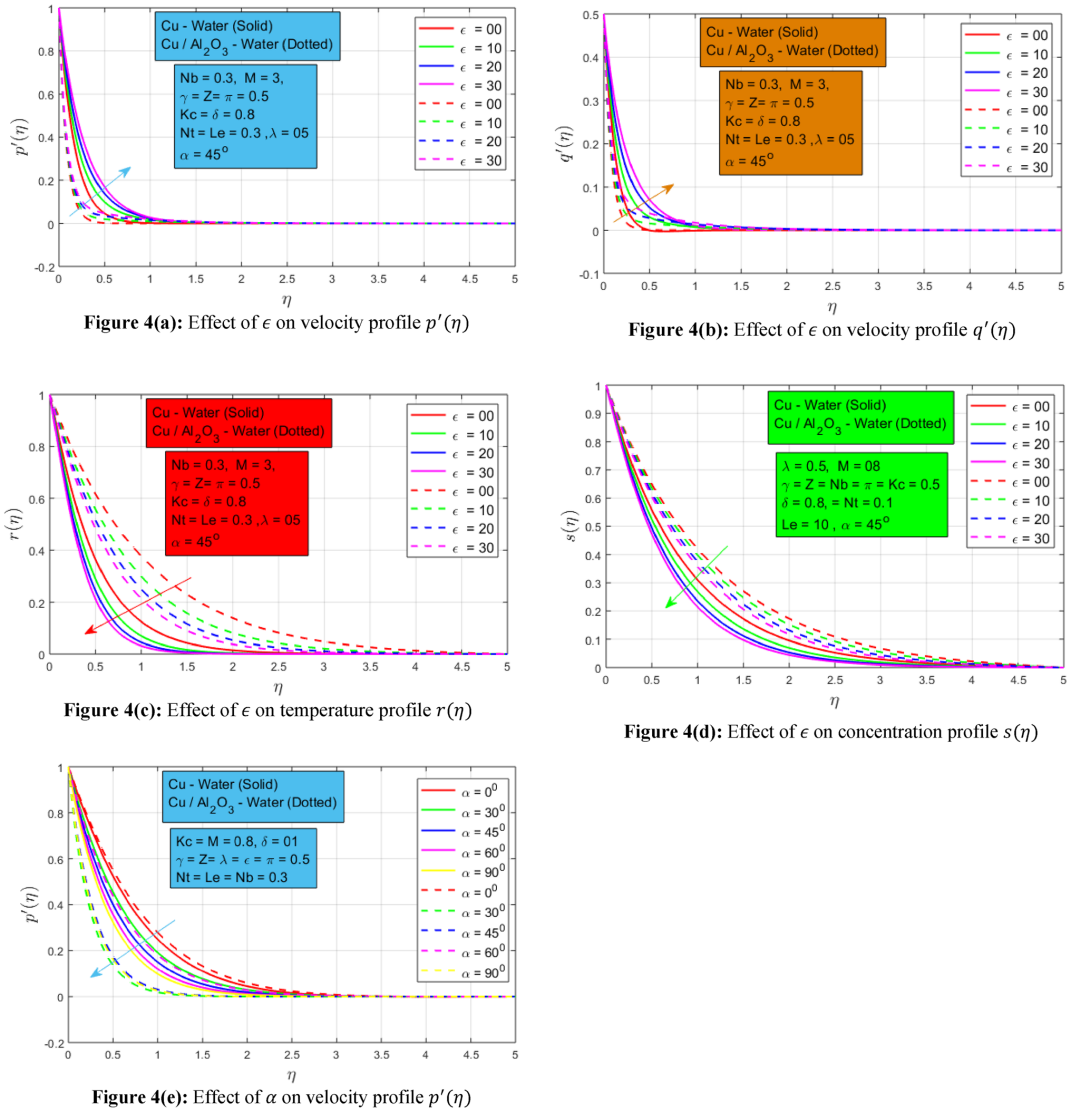
(**a**) Effect of $\epsilon$ on velocity profile ${p}^{^{\prime}}(\eta )$, (**b**) Effect of $\epsilon$ on velocity profile ${q}^{^{\prime}}(\eta )$. (**c**) Effect of $\epsilon$ on temperature profile $r(\eta )$, (**d**) Effect of $\epsilon$ on concentration profile $s(\eta )$, (**e**) Effect of $\alpha$ on velocity profile ${p}^{^{\prime}}(\eta )$.

The following Figs. 4a–d shows the change of mixed convection constraint $\epsilon$ on velocity, temperature, and concentration profiles ${p}^{^{\prime}}\left(\eta \right), {q}^{^{\prime}}\left(\eta \right), r(\eta )$ and $s(\eta )$ respectively. Both velocities profiles ${p}^{^{\prime}}\left(\eta \right)$ and ${q}^{^{\prime}}\left(\eta \right)$ has a linear relationship with mixed convection constraint. In the non-existence of mixed convection, the nanofluid and hybrid nanofluid move very smoothly. When the mixed convection parameter upsurges to a non-zero value the velocities profiles increase in the same way. Since mixed convection magnifies the buoyancy force and as a result, the velocity profiles increase. The higher momentum boundary layer is noted for nanofluid due to the presence of only a single nanoparticle in a base fluid. Due to the lower density of single nanoparticle nanofluid, it can move easily as compared to hybrid nanoparticle nanofluid. While inverse conduct is noted for temperature profile $r(\eta )$ and concentration profile $s(\eta )$ for the increasing estimates of mixed convection constraint. The temperature and concentration of both profiles decay by strengthening the mixed convection constraint. The buoyancy forces dominate the inertial forces, as a result, the temperature and concentration profile reduce. Moreover, it is interesting to note down in the temperature profile, a wider thermal boundary layer is detected for hybrid nanoparticle nanofluid as associated to the single nanoparticle nanofluid. Figure 4e shows the influence of the angle of inclination of the magnetic field acting on the rotational nanofluid and hybrid nanofluid. This provides a magnificent consequence of velocity profile ${p}^{^{\prime}}(\eta )$. There is a trigonometric "sin" function that is involved in magnetic field strength. As the angle of the inclined magnetic field increases from ${0}^{0}\to {90}^{0},$ the inclined magnetic field strength terms get increasing values and as a result, it helps the nanofluid to reduce the speed because the magnitude of resistive force increases.

### Effect of different constraints on temperature and concentration:

**Figure 5:**
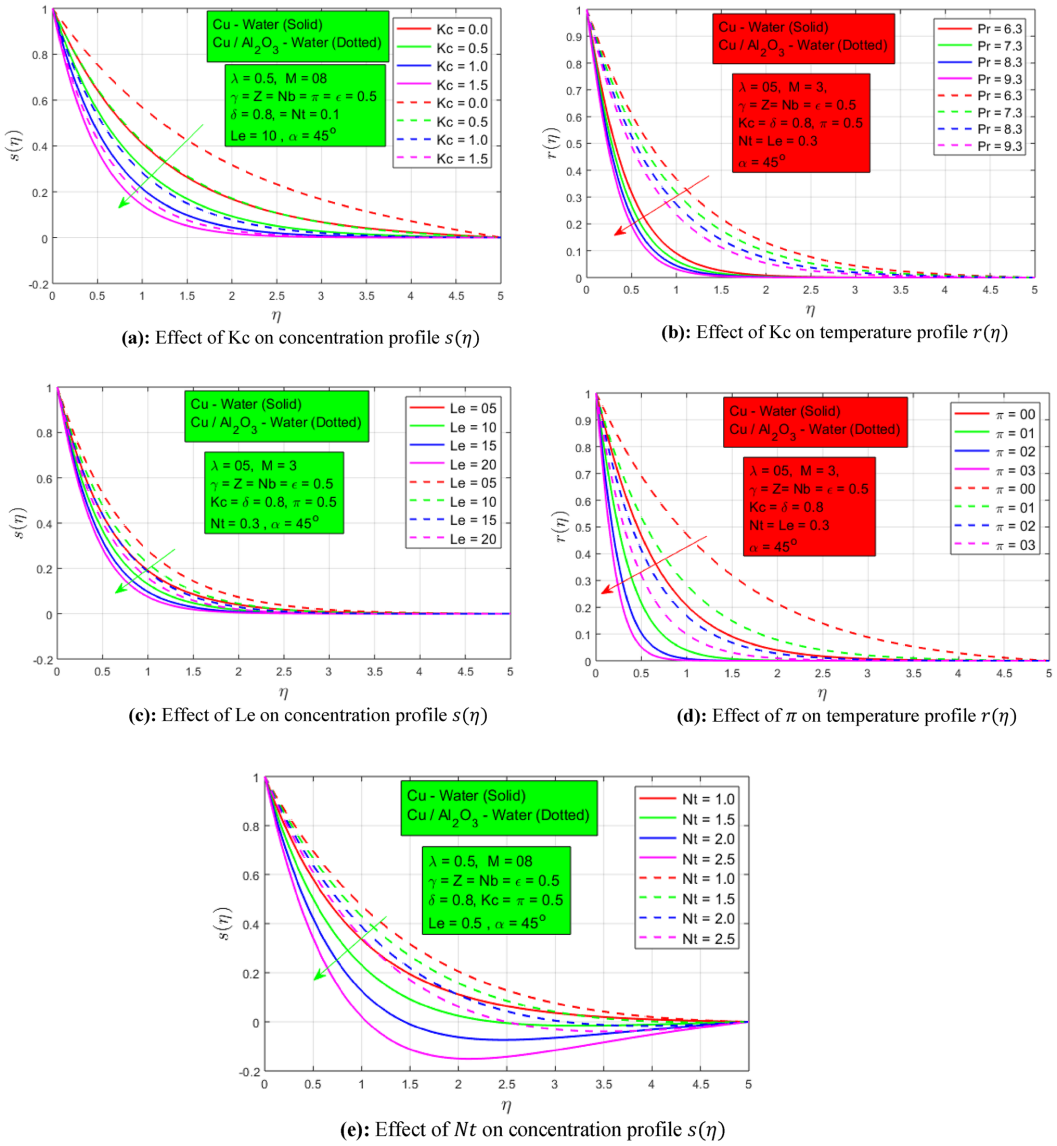
(**a**) Effect of Kc on concentration profile $s(\eta )$. (**b**) Effect of Kc on temperature profile $r(\eta )$, (**c**) Effect of Le on concentration profile $s(\eta )$. (**d**) Effect of $\pi$ on temperature profile $r(\eta )$. (**e**) Effect of $Nt$ on concentration profile $s(\eta )$.

Figure 5a shows the impression of chemical reaction on the concentration profile. In the absence of chemical response, the highest concentration border layer is noted for hybrid nanofluid. When the chemical reaction constraint begins to rise, the concentration profiles start to decay because when the chemical reaction rate increases, a higher volume fraction of nanoparticles undergoes the chemical reaction. A maximum decay is observed for the single-particle nanofluid. So, a consistent mass transfer rate is examined for the hybrid nanoliquid. Figure 5b indicates the change in Prandtl number on the temperature profile. As the significance of the Prandtl number boosts the temperature boundary layer declines since the thermal diffusivity decreases with a rise in the Prandtl number. A higher temperature boundary layer is observed for hybrid nanofluid as associated with nanofluid. Figure 5c shows the influence of Lewis number on the concentration profile. It is defined as the proportion of thermal diffusivity to mass diffusivity. So, the concentration profile decreases once the Lewis number rises. The most important factor of this research is the thermal radiation constraint whose influence on the temperature profile is indicated in Fig. 5d. In the absence of thermal radiation, maximum thermal boundary layer wideness is notable for hybrid nanoliquids over the permeable stretchable surface. When the value of the thermal radiation parameter $\pi$ increases, a sudden fall in the thermal boundary layer is seen for a single nanoparticle nanofluid as compared to the hybrid nanofluid. This shows the efficiency of the hybrid nanofluid for a higher heat transfer amount and reduced skin friction. Figure 5e describes the influence of the thermophoresis parameter $Nt$ on the concentration profile. When the thermophoresis parameter $Nt$ increases, the concentration profile decays due to the inverse relation of $Nt$ with the concentration profile. Inconsistent behavior can be seen for nanofluid and consistency for hybrid nanofluid which shows the efficiency of hybrid nanofluid as associated with the single nanoparticle nanofluid.

### Numerical outcomes for skin frictions, Nusselt, and Sherwood number

In this section, numerical outputs for skin frictions ${Cf}_{x}$, ${Cf}_{y}$, Nusselt, and Sherwood number $s$ are presented. Tables 4 and 5 indicate the numerical outcomes of nanofluid and hybrid nanofluid for different values of parameters. Reduced skin friction and increasing behavior of Nusselt and Sherwood numbers are noted for escalating values of the stretching ratio parameter. Minimum skin friction and the highest Nusselt number are noted in the absence of rotation while it decreases when the rotation of fluid increases. Nusselt number increases when the porosity increases. The angle of the inclined magnetic field has a converse relation with the Nusselt number and the extreme value of the Nusselt number is detected when the axis of rotation and magnetic field are parallel. When the radiation constraint and Prandtl number increases, the Nusselt and Sherwood number both increase. Lewis number, thermophoresis, chemical reaction, and Brownian motion constraint have a negligible effect on skin frictions and Nusselt number but have increasing relation with Sherwood number. Higher Nusselt number and Sherwood number for hybrid nanofluid under the increasing values of thermal radiation.

**Table 4:** Numerical outcomes of single nanoparticle nanofluid for different parameters.

$\gamma$	$\lambda$	$Z$	$M$	$\alpha$	$\pi$	$Pr$	$Le$	$Nt$	$Nb$	$Kc$	${Cf}_{x}$	${Cf}_{y}$	${Nu}_{x}$	${Sh}_{x}$
$0.9$	$5$	$0.5$	$0.3$	${45}^{0}$	$0.5$	$6.3$	$05$	$0.1$	$0.5$	$0.5$	$-1.27171$	$-5.84621$	$1.77692$	$1.45564$
$1.0$											$-1.06746$	$-6.13323$	$1.93212$	$1.49735$
$1.1$											$-0.863848$	$-6.42358$	$2.0792$	$1.53643$
$0.5$	$00$	$0.5$	$0.3$	${45}^{0}$	$0.5$	$6.3$	$05$	$0.1$	$0.5$	$0.5$	$-1.74899$	$-0.545345$	$2.50961$	$1.59187$
	$01$										$-1.5807$	$-1.87334$	$2.17488$	$1.51198$
	$02$										$-1.68379$	$-2.82432$	$1.77526$	$1.42861$
$0.5$	$05$	$00$	$0.3$	${45}^{0}$	$0.5$	$6.3$	$05$	$0.1$	$0.5$	$0.5$	$-1.99491$	$-4.76138$	$1.04238$	$1.24271$
		$0.5$									$-2.09206$	$-4.71868$	$1.08024$	$1.25088$
		$1.0$									$-2.1898$	$-4.67901$	$1.11506$	$1.25802$
$0.5$	$05$	$00$	$00$	${45}^{0}$	$0.5$	$6.3$	$05$	$0.1$	$0.5$	$0.5$	$-1.9536$	$-4.78056$	$1.02532$	$1.23889$
			$05$								$-9.517$	$-5.88903$	$0.911947$	$1.12489$
			$10$								$-19.3165$	$-10.2274$	$0.581224$	$0.935991$
$0.5$	$0.5$	$0.5$	$0.8$	${0}^{0}$	$0.5$	$6.3$	$0.3$	$0.3$	$0.3$	$0.8$	$-1.9536$	$-4.78056$	$1.06268$	$0.913229$
				${30}^{0}$							$-2.10108$	$-4.71489$	$1.12269$	$0.919086$
				${45}^{0}$							$-2.24988$	$-4.65621$	$1.17533$	$0.92419$
				${60}^{0}$							$-2.3994$	$-4.60452$	$1.22029$	$0.928539$
				${90}^{0}$							$-2.54905$	$-4.55966$	$1.25776$	$0.932164$
$0.5$	$05$	$0.5$	$0.3$	${45}^{0}$	$0.0$	$6.3$	$05$	$0.1$	$0.5$	$0.5$	$-1.99491$	$-4.76138$	$3.70957$	$1.24271$
					$2.0$						$-1.99491$	$-4.76138$	$2.32195$	$1.23587$
					$4.0$						$-1.99491$	$-4.76138$	$1.86395$	$1.2338$
$0.5$	$05$	$0.5$	$0.3$	${45}^{0}$	$0.5$	$7.3$	$05$	$0.1$	$0.5$	$0.5$	$-1.99491$	$-4.76138$	$1.21089$	$1.24605$
						$8.3$					$-1.99491$	$-4.76138$	$1.38678$	$1.24964$
						$9.3$					$-1.99491$	$-4.76138$	$1.568$	$1.25344$
$0.5$	$05$	$0.5$	$0.3$	${45}^{0}$	$0.5$	$6.3$	$10$	$0.1$	$0.5$	$0.5$	$-1.99491$	$-4.76138$	$1.02224$	$1.59666$
							$15$				$-1.99491$	$-4.76138$	$1.01113$	$1.85439$
							$20$				$-1.99491$	$-4.76138$	$1.00378$	$2.06212$
$0.5$	$05$	$0.5$	$0.3$	${45}^{0}$	$0.5$	$6.3$	$05$	$00$	$0.5$	$0.5$	$-1.99491$	$-4.76138$	$1.07715$	$1.23143$
								$03$			$-1.99491$	$4.76138$	$0.47619$	$1.26056$
								$06$			$-1.99491$	$4.76138$	$0.280558$	$1.16311$
$0.5$	$05$	$0.5$	$0.3$	${45}^{0}$	$0.5$	$6.3$	$05$	$0.1$	$0.7$	$0.5$	$-1.99491$	$-4.76138$	$0.95685$	$1.24453$
									$0.8$		$-1.99491$	$-4.76138$	$0.916201$	$1.24494$
									$0.9$		$-1.99491$	$-4.76138$	$0.876938$	$1.24506$
$0.5$	$05$	$0.5$	$0.3$	${45}^{0}$	$0.5$	$6.3$	$05$	$0.1$	$0.5$	$05$	$-1.99491$	$-4.76138$	$0.968205$	$5.15805$
										$10$	$-1.99491$	$-4.76138$	$0.95203$	$10.0714$
										$15$	$-1.99491$	$-4.76138$	$0.945936$	$15.0475$

**Table 5:** Numerical outcomes of hybrid nanoparticle nanofluid for different parameter.

$\gamma$	$\lambda$	$Z$	$M$	$\alpha$	$\pi$	$Pr$	$Le$	$Nt$	$Nb$	$Kc$	${Cf}_{x}$	${Cf}_{y}$	${Nu}_{x}$	${Sh}_{x}$
$0.9$	$5$	$0.5$	$0.3$	${45}^{0}$	$0.5$	$6.3$	$05$	$0.1$	$0.5$	$0.5$	$-1.30977$	$-4.84593$	$2.67373$	$1.48781$
$1.0$											$-1.15104$	$-5.10257$	$2.87171$	$1.52715$
$1.1$											$-0.992821$	$-5.36196$	$3.05844$	$1.56418$
$0.5$	$00$	$0.5$	$0.3$	${45}^{0}$	$0.5$	$6.3$	$05$	$0.1$	$0.5$	$0.5$	$-1.87119$	$-0.748302$	$3.24348$	$1.55916$
	$01$										$-1.71084$	$-1.56928$	$2.99208$	$1.5121$
	$02$										$-1.55124$	$-1.40626$	$2.62454$	$1.47942$
$0.5$	$05$	$00$	$0.3$	${45}^{0}$	$0.5$	$6.3$	$05$	$0.1$	$0.5$	$0.5$	$-1.86586$	$-3.86583$	$1.70799$	$1.29306$
		$0.5$									$-1.94748$	$-3.83689$	$1.74894$	$1.29807$
		$1.0$									$-2.02921$	$-3.81044$	$1.78495$	$1.3023$
$0.5$	$05$	$00$	$00$	${45}^{0}$	$0.5$	$6.3$	$05$	$0.1$	$0.5$	$0.5$	$-1.6217$	$-3.96838$	$1.55311$	$1.27254$
			$05$								$-21.3547$	$-11.0332$	$0.69446$	$0.884768$
			$10$								$-42.8152$	$-21.5857$	$0.512811$	$0.744221$
$0.5$	$0.5$	$0.5$	$0.8$	${0}^{0}$	$0.5$	$6.3$	$0.3$	$0.3$	$0.3$	$0.8$	$-1.6217$	$-3.96838$	$1.6146$	$0.926378$
				${30}^{0}$							$-3.81472$	$-3.69943$	$1.96095$	$0.951212$
				${45}^{0}$							$-3.30537$	$-3.65928$	$2.00661$	$0.954569$
				${60}^{0}$							$-1.84435$	$-3.87388$	$1.76145$	$0.936731$
				${90}^{0}$							$-3.46211$	$-3.66696$	$1.99577$	$0.953781$
$0.5$	$05$	$0.5$	$0.3$	${45}^{0}$	$0.0$	$6.3$	$05$	$0.1$	$0.5$	$0.5$	$-1.86586$	$-3.86583$	$5.02414$	$1.29306$
					$2.0$						$-1.86586$	$-3.86583$	$3.24482$	$1.28519$
					$4.0$						$-1.86586$	$-3.86583$	$2.48104$	$1.28216$
$0.5$	$05$	$0.5$	$0.3$	${45}^{0}$	$0.5$	$7.3$	$05$	$0.1$	$0.5$	$0.5$	$-1.86586$	$-3.86583$	$1.94337$	$1.65068$
						$8.3$					$-1.86586$	$-3.86583$	$2.22159$	$1.65422$
						$9.3$					$-1.86586$	$-3.86583$	$2.50006$	$1.65795$
$0.5$	$05$	$0.5$	$0.3$	${45}^{0}$	$0.5$	$6.3$	$10$	$0.1$	$0.5$	$0.5$	$-1.86586$	$-3.86583$	$1.66866$	$1.6474$
							$15$				$-1.86586$	$-3.86583$	$1.64685$	$1.90337$
							$20$				$-1.86586$	$-3.86583$	$1.63229$	$2.10923$
$0.5$	$05$	$0.5$	$0.3$	${45}^{0}$	$0.5$	$6.3$	$05$	$00$	$0.5$	$0.5$	$-1.86586$	$-3.86583$	$1.77353$	$1.27844$
								$03$			$-1.86586$	$-3.86583$	$0.712169$	$1.30163$
								$06$			$-1.86586$	$-3.86583$	$0.40483$	$1.20479$
$0.5$	$05$	$0.5$	$0.3$	${45}^{0}$	$0.5$	$6.3$	$05$	$0.1$	$0.7$	$0.5$	$-1.86586$	$-3.86583$	$1.5587$	$1.2953$
									$0.8$		$-1.86586$	$-3.86583$	$1.48782$	$1.29576$
									$0.9$		$-1.86586$	$-3.86583$	$1.41943$	$1.29583$
$0.5$	$05$	$0.5$	$0.3$	${45}^{0}$	$0.5$	$6.3$	$05$	$0.1$	$0.5$	$05$	$-1.86586$	$-3.86583$	$1.56006$	$5.16945$
										$10$	$-1.86586$	$-3.86583$	$1.52372$	$10.0781$
										$15$	$-1.86586$	$-3.86583$	$1.50991$	$15.0535$

## Conclusions

The current research investigates three-dimensional, rotating, incompressible, nanofluid, and hybrid nanofluid flow above the permeable stretchable surface for the heat and mass transmission rate. The governing equations are tackled at MATLAB through the bvp-4c algorithm after employing the similarity transformation. The major outcomes of the present study are stated below:

1. The velocity profiles decline with a rise in rotation, magnetic field, porosity, and increasing angle between the axis of rotation and the horizontal axis while increasing for mixed convection and stretching ratio parameter.
2. Prandtl number and thermal radiation, thermal slip, and stretching ratio parameters have decreasing effects on the temperature profile.
3. The concentration profile decreases under the increasing influence of thermophoresis and but has an increasing relation with the rotation parameter when it increases.
4. A maximum Nusselt and Sherwood number is noted in the absence of rotation and porosity of the medium.
5. The highest heat and mass transfer rate is noted when the inclined magnetic field and axis rotation are parallel to each other.
6. Thermophoresis and chemical reaction parameters increase the mass transfer rate when it increases.
7. Thermal radiation rapidly increases the heat and mass transmission when it increases.
8. Higher heat transfer rates and reduced skin friction are noted for hybrid nanofluid.

## Data Availability

All data generated or analyzed during this study are included in this published article.

## List of symbols

$${\omega }^{*}$$: Angular velocity
$${C}_{p}$$: Specific heat
${\phi }_{1},{\phi }_{2}$: Volume fraction for nanoparticles
${A}_{1}, {A}_{2},{A}_{3}, {A}_{4}$: Constants for single nanoparticle nanofluid
$T,C$: Temperature and concentration
$$Z$$: Porosity parameter
$a,b$: Stretching rate along x and y axis
$$Pr$$: Prandtl number
$\pi$: Thermal radiation parameter
$$\alpha$$: Inclination angle
$${B}_{0}$$: Magnetic field
${N}_{t}, {N}_{b}$: Thermophoresis and Brownian motion parameter
$${q}_{w}$$: Heat flux
*R*: Reynolds number
*Kc*: Chemical reaction
$${g}^{*}$$: Gravitational acceleration
$\gamma$: Stretching ratio parameter
$$k$$: Thermal conductivity
${Nu}_{x}$: Nusselt number coefficient
$$\eta$$: Similarity variable
$u,v$*, w*: Velocity components in $\mathrm{x},\mathrm{y},\mathrm{z}$ direction
$${Cf}_{x}, {Cf}_{y}$$: Skin frictions
$${p}^{^{\prime}}, {q}^{^{\prime}}$$: Dimensionless velocity
${B}_{1}, {B}_{2},{B}_{3}, {B}_{4}$: Constants for double nanoparticle nanofluid
*Le*: Lewis number
$${D}_{B}$$: Brownian motion
*V*: Velocity field
${\beta }_{t}$: Thermal volumetric coefficient
$${v}_{f}$$: Kinematic viscosity
$${\mu }_{f}$$: Dynamic viscosity
$r,s$: Temperature and concentration profile
*3D*: Three dimensional

## Greek symbols

$${\alpha }_{1}$$: Temperature diffusivity
$$\uplambda$$: Rotational velocity
$$\rho$$: Density
$$\sigma$$: Electrical conductivity
$${\tau }_{w}$$: Shear stress
$\epsilon$: Mixed convection parameter
