# Author Correction: Effect of inclined magnetic field on radiative heat and mass transfer in chemically reactive hybrid nanofluid flow due to dual stretching

**DOI:** 10.1038/s41598-025-17824-2

**Published:** 2025-10-06

**Authors:** Mubashar Arshad, Fahad M. Alharbi, Ali Hassan, Qusain Haider, Abdullah Alhushaybari, Sayed M. Eldin, Zubair Ahmad, Laila A. Al‑Essa, Ahmed M. Galal

**Affiliations:** 1 Department of Mathematics, University of Gujrat, Gujrat, 50700 Pakistan; 2 Department of Mathematics, Al‑Qunfudah University College, Umm Al-Qura University, Mecca, Saudi Arabia; 3 Department of Mathematics, College of Science, Taif University, P.O. Box 11099, 21944 Taif, Saudi Arabia; 4 Center of Research, Faculty of Engineering, Future University in Egypt, New Cairo, 11835 Egypt; 5 Unit of Bee Research and Honey Production, Faculty of Science, King Khalid University, P.O. Box 9004, 61413 Abha, Saudi Arabia; 6 Applied College, Mahala Campus, King Khalid University, P.O. Box 9004, 61413 Abha, Saudi Arabia; 7 Department of Mathematical Sciences, College of Science, Princess Nourah Bint Abdulrahman University, P.O. Box 84428, 11671 Riyadh, Saudi Arabia; 8 Department of Mechanical Engineering, College of Engineering in Wadi Alddawasir, Prince Sattam Bin Abdulaziz University, Wadi Alddawasir, Saudi Arabia; 9 Production Engineering and Mechanical Design Department, Faculty of Engineering, Mansoura University, P.O. 35516, Mansoura, Egypt

Correction to: *Scientific Reports* 10.1038/s41598-023-34871-9, published online 15 May 2023

The original Article contained errors. Due to an error during formulation and simplification of Eqs. (6) and (7), which are derived from a generalized Navier–Stokes framework that includes body force terms, the gravitational force was retained. This term has now been removed.

The original Eq. (6):

$$\left( {uu_{x} + vu_{y} + wu_{z} - 2\omega^{*} v} \right) = \frac{{\mu_{hnf} }}{{\rho_{hnf} }}\left( {u_{xx} + u_{yy} + u_{zz} } \right) + \frac{{g^{*} \left( {\rho B_{t} } \right)_{hnf} }}{{\rho_{hnf} }}\left( {T - T_{\infty } } \right) - \frac{{\sigma_{hnf} }}{{\rho_{hnf} }}B_{0}^{2} \sin^{2} \left( \alpha \right)u - \frac{{\mu_{hnf} }}{{\rho_{hnf} }}\frac{u}{{k_{o} }},$$

Now reads:

$$\left( {u u_{x} + v u_{y} + w u_{z} - 2\omega^{*} v} \right) = \frac{{\mu_{hnf} }}{{\rho_{hnf} }}\left( {u_{xx} + u_{yy} + u_{zz} } \right) - \frac{{\sigma_{hnf} }}{{\rho_{hnf} }}B_{0}^{2} \sin^{2} \left( \alpha \right)u - \frac{{\mu_{hnf} }}{{\rho_{hnf} }}\frac{u}{{k_{o} }},$$

The original Eq. (7):

$$\left( {uv_{x} + vv_{y} + wv_{z} - 2\omega^{*} u} \right) = \frac{{\mu_{hnf} }}{{\rho_{hnf} }}\left( {v_{xx} + v_{yy} + v_{zz} } \right) + \frac{{g^{*} \left( {\rho B_{t} } \right)_{hnf} }}{{\rho_{hnf} }}\left( {T - T_{\infty } } \right) - \frac{{\sigma_{hnf} }}{{\rho_{hnf} }}B_{0}^{2} \sin^{2} \left( \alpha \right)v - \frac{{\mu_{hnf} }}{{\rho_{hnf} }}\frac{v}{{k_{o} }},$$

Now reads:

$$\left( {u v_{x} + v v_{y} + w v_{z} - 2\omega^{*} u} \right) = \frac{{\mu_{hnf} }}{{\rho_{hnf} }}\left( { v_{xx} + v_{yy} + v_{zz} } \right) - \frac{{\sigma_{hnf} }}{{\rho_{hnf} }}B_{0}^{2} \sin^{2} \left( \alpha \right)v - \frac{{\mu_{hnf} }}{{\rho_{hnf} }}\frac{v}{{k_{o} }},$$

This error also requires changes to the Eqs. (17), (18), and (21), as these are derived from (6) and (7).

The original Eq. (17):

$$p^{\prime \prime \prime } \left( \eta \right) = B_{1} *\left\{ {p^{\prime } \left( \eta \right)^{2} \left( {p^{\prime } \left( \eta \right) + q^{\prime } \left( \eta \right)} \right) - 2\lambda \delta q^{\prime } \left( \eta \right) + Zp^{\prime } \left( \eta \right) - \epsilon_{x} rB_{4} + M^{2} \sin^{2} \left( \alpha \right)p^{\prime } B_{5} } \right\}*K_{2}$$

Now reads:

$$p^{\prime \prime \prime } \left( \eta \right) = B_{1} *\left\{ {p^{\prime } \left( \eta \right)^{2} \left( {p^{\prime } \left( \eta \right) + q^{\prime } \left( \eta \right)} \right) - 2\lambda \delta q^{\prime } \left( \eta \right) + Zp^{\prime } \left( \eta \right) + M^{2} \sin^{2} \left( \alpha \right)p^{\prime } B_{5} } \right\}*K_{2}$$

The original Eq. (18):

$$q^{\prime \prime \prime } \left( \eta \right) = B_{1} *\left\{ {q^{\prime } \left( \eta \right)^{2} pv\left( \eta \right) + q^{\prime } \left( \eta \right) - 2\frac{\lambda }{\delta }p^{\prime } \left( \eta \right) + Zq^{\prime } \left( \eta \right) - \epsilon_{y} rB_{4} + M^{2} \sin^{2} \left( \alpha \right)q^{\prime } B_{5} } \right\}*K_{2}$$

Now reads:

$$q^{\prime \prime \prime } \left( \eta \right) = B_{1} *\left\{ {q^{\prime } \left( \eta \right)^{2} \left( {p^{\prime } \left( \eta \right) + q^{\prime } \left( \eta \right)} \right) - 2\frac{\lambda }{\delta } p^{\prime } \left( \eta \right) + Z q^{\prime } \left( \eta \right) + M^{2} \sin^{2} \left( \alpha \right)q^{\prime } B_{5} } \right\}*K_{2}$$

The original Eq. (21):

$$\begin{gathered} \lambda = \frac{{\omega^{*} }}{a}, \quad \delta = \frac{y}{x},\quad Z = \frac{{\mu_{hnf} }}{{a\rho_{hnf} k_{o} }},\quad \epsilon_{x} = \frac{{Gr_{x} }}{{{\text{Re}}_{x}^{2} }}, \quad \epsilon_{y} = \frac{{Gr_{y} }}{{{\text{Re}}_{y}^{2} }},\quad M = \sqrt {\frac{{\sigma_{f} B_{0}^{2} }}{{a\rho_{f} }}} ,\quad \Pr = \frac{{v_{f} }}{{k_{f} }}, \quad \pi = \frac{{4\sigma^{*} T_{\infty }^{3} }}{{k_{1} k_{f} }},\quad Le = \frac{{D_{B} }}{{v_{f} }},\quad \tau = \frac{{\left( {\rho Cp} \right)_{s} }}{{\left( {\rho Cp} \right)_{f} }}, \hfill \\ N_{t} = \frac{{\left( {\rho Cp} \right)_{s} D_{B} \left( {C_{w} - C_{\infty } } \right)}}{{\left( {\rho Cp} \right)_{f} v_{f} }},\quad N_{b} = \frac{{\left( {\rho Cp} \right)_{s} D_{T} \left( {T_{w} - T_{\infty } } \right)}}{{\left( {\rho Cp} \right)_{f} T_{\infty } v_{f} }},\quad K_{c} = \frac{{K_{r} }}{a}. \hfill \\ \end{gathered}$$

Now reads:

$$\begin{gathered} \lambda = \frac{{\omega^{*} }}{a},\delta = \frac{y}{x},Z = \frac{{\mu_{hnf} }}{{a\rho_{hnf} k_{o} }},M = \sqrt {\frac{{\sigma_{f} B_{0}^{2} }}{{a\rho_{f} }}} ,\Pr = \frac{{v_{f} }}{{k_{f} }},\pi = \frac{{4\sigma^{*} T_{\infty }^{3} }}{{k_{1} k_{f} }},Le = \frac{{D_{B} }}{{v_{f} }},\tau = \frac{{\left( {\rho Cp} \right)_{s} }}{{\left( {\rho Cp} \right)_{f} }}, \hfill \\ N_{t} = \frac{{\left( {\rho Cp} \right)_{s} D_{B} \left( {C_{w} - C_{\infty } } \right)}}{{\left( {\rho Cp} \right)_{f} v_{f} }},N_{b} = \frac{{\left( {\rho Cp} \right)_{s} D_{T} \left( {T_{w} - T_{\infty } } \right)}}{{\left( {\rho Cp} \right)_{f} T_{\infty } v_{f} }},K_{c} = \frac{{K_{r} }}{a} \hfill \\ \end{gathered}$$

Due to this error, numerical values in Tables 4 and 5 have changed. The original Tables 4 and 5 and accompanying legends appear below as Tables 1 and 2.

**Table 1:** Numerical outcomes of single nanoparticle nanofluid for different parameters.

*γ*	*λ*	*Z*	*ε*	*M*	*α*	*π*	*Pr*	*Le*	*Nt*	*Nb*	*Kc*	*Cf*_*x*_	*Cf*_*y*_	*Nu*_*x*_	*Sh*_*x*_
0.9	5	0.5	0.5	0.3	45°	0.5	6.3	05	0.1	0.5	0.5	− 0.66635	− 5.11895	4.92841	1.43954
1.0												− 0.41465	− 5.40745	5.15842	1.45668
1.1												− 0.16347	− 5.69962	5.37524	1.47315
0.5	00	0.5	0.5	0.3	45°	0.5	6.3	05	0.1	0.5	0.5	− 1.63057	− 0.408852	5.09938	1.47146
	01											− 1.35134	− 1.51853	4.86746	1.47669
	02											− 1.38521	− 2.33791	4.58522	1.44866
0.5	05	00	0.5	0.3	45°	0.5	6.3	05	0.1	0.5	0.5	− 1.56568	− 4.01668	3.79057	1.36584
		0.5										− 1.67181	− 3.98974	3.81111	1.36327
		1.0										− 1.77825	− 3.96402	3.82727	1.36082
0.5	05	00	00	0.3	45°	0.5	6.3	05	0.1	0.5	0.5	− 1.83082	− 4.05492	3.73971	1.35147
			01									− 1.51648	− 3.92227	3.87715	1.37418
			02									− 1.21534	− 3.78735	3.99809	1.39384
0.5	05	00	0.5	00	45°	0.5	6.3	05	0.1	0.5	0.5	− 1.62639	− 4.00051	3.80291	1.36422
				05								− 9.40586	− 5.64041	2.62335	1.05891
				10								− 19.2573	− 10.0976	1.54102	0.86689
0.5	0.5	0.5	0.5	0.8	0°	0.5	6.3	0.3	0.3	0.3	0.8	− 1.30397	− 0.96499	5.03399	1.16886
					30°							− 1.65745	− 1.07217	4.93271	1.15533
					45°							− 1.96241	− 1.18232	4.83962	1.14397
					60°							− 2.23195	− 1.28877	4.75467	1.13435
					90°							− 2.47536	− 1.39003	4.67661	1.12604
0.5	05	0.5	0.5	0.3	45°	0.0	6.3	05	0.1	0.5	0.5	− 1.64161	− 3.99444	9.33755	1.33908
						2.0						− 1.72006	− 3.99117	23.0151	1.42702
						4.0						− 1.74731	− 3.99885	32.0469	1.49335
0.5	05	0.5	0.5	0.3	45°	0.5	7.3	05	0.1	0.5	0.5	− 1.68193	− 3.98832	4.26657	1.37346
							8.3					− 1.69066	− 3.98821	4.69223	1.38333
							9.3					− 1.69823	− 3.98865	5.09217	1.39285
0.5	05	0.5	0.5	0.3	45°	0.5	6.3	10	0.1	0.5	0.5	− 1.67181	− 3.98941	3.81111	1.36327
15												− 1.67181	− 3.98944	3.81111	1.67674
20												− 1.67181	− 3.98945	3.81111	1.90002
0.5	05	0.5	0.5	0.3	45°	0.5	6.3	05	00	0.5	0.5	− 1.67181	− 3.98947	3.81111	1.29326
									03			− 1.67181	− 3.98948	3.81111	3.39361
									06			− 1.67181	− 3.98948	3.81111	5.49397
0.5	05	0.5	0.5	0.3	45°	0.5	6.3	05	0.1	0.7	0.5	− 1.67181	− 3.98948	3.81111	1.39128
										0.8		− 1.67181	− 3.98947	3.81111	1.40528
										0.9		− 1.67181	− 3.98943	3.81111	1.41928
0.5	05	0.5	0.5	0.3	45°	0.5	6.3	05	0.1	0.5	05	− 1.67181	− 3.98942	3.81111	5.14727
											10	− 1.67181	− 3.98941	3.81111	10.0610
											15	− 1.67181	− 3.98944	3.81111	15.0399

**Table 2:** Numerical outcomes of hybrid nanoparticle nanofluid for different parameter.

*γ*	*λ*	*Z*	*ε*	*M*	*α*	*π*	*Pr*	*Le*	*Nt*	*Nb*	*Kc*	*Cf*_*x*_	*Cf*_*y*_	*Nu*_*x*_	*Sh*_*x*_
0.9	5	0.5	0.5	0.3	45°	0.5	6.3	05	0.1	0.5	0.5	− 0.43183	− 2.18251	8.79526	1.43068
1.0												− 0.33186	− 2.31401	9.19781	1.44593
1.1												− 0.23213	− 2.44702	9.57884	1.46064
0.5	00	0.5	0.5	0.3	45°	0.5	6.3	05	0.1	0.5	0.5	− 0.90985	− 0.33342	8.80656	1.43247
	01											− 0.79227	− 0.67825	8.53503	1.44253
	02											− 0.76710	− 0.98631	8.13384	1.42848
0.5	05	00	0.5	0.3	45°	0.5	6.3	05	0.1	0.5	0.5	− 0.78680	− 1.67519	6.85763	1.36639
		0.5										− 0.83178	− 1.66646	6.87709	1.36341
		1.0										− 0.87671	− 1.65882	6.89059	1.36021
0.5	05	00	00	0.3	45°	0.5	6.3	05	0.1	0.5	0.5	− 0.88886	− 1.69231	6.76566	1.35382
			01									− 0.77586	− 1.64047	6.98128	1.37236
			02									− 0.66702	− 1.58814	7.17191	1.38872
0.5	05	00	0.5	00	45°	0.5	6.3	05	0.1	0.5	0.5	− 1.36585	− 3.32792	4.56164	1.37126
				05								− 21.3205	− 10.9546	1.47417	0.81395
				10								− 42.7979	− 21.5463	0.91983	0.69608
0.5	0.5	0.5	0.5	0.8	0°	0.5	6.3	0.3	0.3	0.3	0.8	− 0.55826	− 0.41487	9.05095	1.15121
					30°							− 2.07246	− 1.06463	6.99015	1.04524
					45°							− 1.80146	− 0.93497	7.35539	1.06017
					60°							− 0.81363	− 0.49779	8.72787	1.12908
					90°							− 1.88623	− 0.97533	7.23928	1.05535
0.5	05	0.5	0.5	0.3	45°	0.0	6.3	05	0.1	0.5	0.5	− 0.82410	− 1.66704	12.2541	1.34692
						2.0						− 0.84583	− 1.66762	24.4276	1.40833
						4.0						− 0.85513	− 1.67012	33.1092	1.45769
0.5	05	0.5	0.5	0.3	45°	0.5	7.3	05	0.1	0.5	0.5	− 0.83532	− 1.66648	7.68265	1.37258
							8.3					− 0.83837	− 1.66663	8.43683	1.38147
							9.3					− 0.84101	− 1.66688	9.14676	1.39008
0.5	05	0.5	0.5	0.3	45°	0.5	6.3	10	0.1	0.5	0.5	− 0.83178	− 1.66646	6.87709	1.36341
								15				− 0.83178	− 1.66646	6.87709	1.67527
								20				− 0.83178	− 1.66646	6.87709	1.89803
0.5	05	0.5	0.5	0.3	45°	0.5	6.3	05	00	0.5	0.5	− 0.83178	− 1.66646	6.87709	1.30175
									03			− 0.83178	− 1.66646	6.87709	3.15162
									06			− 0.83178	− 1.66646	6.87709	5.00156
0.5	05	0.5	0.5	0.3	45°	0.5	6.3	05	0.1	0.7	0.5	− 0.83178	− 1.66646	6.87709	1.38807
										0.8		− 0.83178	− 1.66646	6.87709	1.40041
										0.9		− 0.83178	− 1.66646	6.87709	1.41274
0.5	05	0.5	0.5	0.3	45°	0.5	6.3	05	0.1	0.5	05	− 0.83178	− 1.66646	6.87709	5.14607
											10	− 0.83178	− 1.66646	6.87709	10.05925
											15	− 0.83178	− 1.66646	6.87709	15.03831

The original Article has been corrected.

.

.

